# Effect of Antiviral Therapy During Pregnancy on Natural Killer Cells in Pregnant Women With Chronic HBV Infection

**DOI:** 10.3389/fimmu.2022.893628

**Published:** 2022-05-23

**Authors:** Fuchuan Wang, Si Xie, Chongping Ran, Hongxiao Hao, Tingting Jiang, Wen Deng, Xiaoyue Bi, Yanjie Lin, Liu Yang, Fangfang Sun, Zhan Zeng, Yao Xie, Minghui Li, Wei Yi

**Affiliations:** ^1^Department of Gynecology and Obstetrics, Beijing Ditan Hospital, Capital Medical University, Beijing, China; ^2^Division of Hepatology, Hepato-Pancreato-Biliary Center, Beijing Tsinghua Changgung Hospital, School of Clinical Medicine, Tsinghua University, Beijing, China; ^3^Department of Hepatology Division 2, Beijing Ditan Hospital, Capital Medical University, Beijing, China; ^4^Department of Hepatology Division 2, Peking University Ditan Teaching Hospital, Beijing, China

**Keywords:** hepatitis B virus, natural killer cells, chronic hepatitis B, antiviral treatment, postpartum

## Abstract

**Objective:**

To study the effect of antiviral therapy during pregnancy on the frequency of natural killer (NK) cells in peripheral blood of women with HBV DNA positive chronic hepatitis B (CHB).

**Method:**

In total 124 female subjects were divided into four groups: 11 healthy non-pregnant women (Normal group), 26 non-pregnant women in immune tolerance period of chronic hepatitis B virus (HBV) infection (CHB group), 41 pregnant CHB women without antiviral treatment during pregnancy (Untreated group), and 46 pregnant CHB women receiving antiviral treatment during pregnancy (Treated group). The frequency of NK cells in peripheral blood were detected by flow cytometry.

**Result:**

The frequency of NK cells in healthy women [15.30 (12.80, 18.40)] was higher than that in women with HBV infection, but there was no significant statistical difference (*p*=0.436). The frequency of NK cells in CHB group [10.60 (6.00, 18.30)] was higher than those in pregnant CHB women [Untreated: 6.90 (4.89, 10.04), *P*=0.001; Treated: 9.42 (6.55, 14.10), *P*=0.047]. The frequency of NK cells in treated group was significantly higher than that in untreated group (*P* = 0.019). The frequencies of NK cells, CD56^bright^ NK cells and NKp46^dim^ NK cells at 12 and 24 weeks postpartum in the untreated group were increased significantly than those before delivery. In treated group, the frequencies of NK cells, CD56^bright^ NK cells, NKp46^+^ NK cells and NKp46^dim^ NK cells were significantly increased at 6 and 12 weeks than those before delivery. The frequencies of NK cells and CD56^bright^ NK cells postpartum were increased significantly in treated group than those in untreated group. The frequencies of CD56^dim^ NK cells decreased significantly after delivery in treated than those in untreated patients. Alanine aminotransferase (ALT) and aspartate aminotransferase (AST) significantly increased after delivery than those before delivery. The results showed that the postpartum ALT level was weak positive correlated with NKp46^high^ frequency (*r*=0.199) and was weak negative correlated with NKp46^dim^ frequency (*r*= -0.199).

**Conclusion:**

Antiviral treatment during pregnancy could significantly increase the frequency of NK cells postpartum. Postpartum hepatitis may be related to the immune injury caused by change of NK cell frequency and HBV infection.

## Introduction

Mother-to-child transmission of hepatitis B virus (HBV) is the main cause of chronic HBV infection in China (1, 2). For HBV DNA positive pregnant women, antiviral treatment in the third trimester of pregnancy can further reduce the mother-to-child transmission of HBV. Postpartum hepatitis is common in women with chronic hepatitis B (CHB). Our previous research suggests that postpartum hepatitis mainly occurs in pregnant women with HBV DNA > 5.01 log10 IU/ml before delivery, while abnormal postpartum liver function in patients with HBV infection mainly occurs at 3-4 weeks and 9-12 weeks postpartum (3).

Natural killer (NK) cells are one of the important effector cells in antiviral immunity. The amount and function of NK cells decrease in course of chronic HBV infection. To achieve immune recovery by antiviral therapy, recovering of the number and function of NK cells is of great significance. At present, there are few studies on the effect of antiviral therapy on immunological characteristics of postpartum NK cells in CHB patients.

In the present study, we designed a prospective case cohort study in HBV DNA positive CHB pregnant women. We detected the frequencies of NK cell in peripheral blood before delivery and at 6, 12, and 24 weeks after delivery to explore the correlation of NK cells with antiviral therapy in pregnant women with HBV infection.

## Materials and Methods

### Patients

From January 2017 to January 2018, 124 patients from the Department of Gynecology and Obstetrics, Beijing Ditan Hospital, Capital Medical University were included in this study, including 11 healthy cases with hepatitis B surface antigen (HBsAg) negative and normal liver function (Normal group). All patients with hepatitis B virus were immune tolerant patients (HBV DNA> 2.0×10^7^ IU/ml, hepatitis B e antigen and HBsAg continued to be positive more than six months, biochemical examination, imaging examination and other noninvasive examinations showing no clinical characteristics of hepatitis attack). 26 cases of non-pregnant women in immune tolerance period of HBV infection (CHB group), 41 cases of HBV infected pregnant women without antiviral treatment (Untreated group), and 46 pregnant women with HBV infection who received tenofovir disoproxil fumarate (TDF) antiviral therapy at 32 week of pregnancy and stopped immediately after delivery (Treated group). All subjects signed informed consent. The study was approved by the Ethics Committee of Beijing Ditan Hospital, Capital Medical University (JDL-2017-004-01), and registered at clinicaltrials.gov (Clinical registration No: NCT03214302).

Pregnant women have regular prepartum examination. Chronic hepatitis B was diagnosed according to the EASL Clinical Practice Guidelines (4). Exclusion criteria were: combined with hepatitis C virus and other viral infections; autoimmune liver disease, idiopathic cholestasis of pregnancy and other liver diseases; other systemic diseases such as systemic lupus erythematosus, diabetes, hypertensive disorder, etc.

### Blood Specimen Collection

EDTA anticoagulant purple tube was used to collect 2 ml of peripheral venous blood from all pregnant women at the time of prepartum (baseline), 6, 12 and 24 weeks postpartum, while blood samples were taken from non-pregnant women at the time of enrollment. The cells were collected by BD FACS Calibur within 4 h after collection.

### Peripheral Blood NK Cells Were Measured by Flow Cytometry

Anticoagulant whole blood was drawn from the subjects, 3 μL PerCP Mouse anti-Human CD3, 3 μL APC Mouse anti-Human CD56 and 5 μL PE anti-Human CD335 was added to 100 μl anticoagulant whole blood, incubated at room temperature avoid light for 15 min after vortex mixing, and make the same type control, add 2 ml of erythrocyte lysate, vortex and mix well, incubated 3-5 min avoid light at room temperature and centrifuged cells, washed with PBS, resuspended cells, NK cells were detected by flow cytometry. According to the forwards cattering (FSC) and lateral sides cattering (SSC) gates the lymphocyte population, and more than 10000 lymphocytes were obtained from each sample. NK cell image analysis was performed using FlowJo software.

### Relevant Index Detection

HBV serological markers (HBsAg and HBeAg) were measured by chemiluminescent microparticle immunoassay (Architect i2000 analyzer; Abbott Diagnostics, Abbott Park, IL, USA). Serum HBV DNA was tested by real-time quantitative PCR (Piji Co, Ltd, Shenzhen, China) with a detection range of 100 - 2.0×10^9^ IU/ml. Alanine aminotransferase (ALT) and aspartate aminotransferase (AST) level were tested using a Hitachi 7600 fully automatic biochemical analyzer with the ULN set at 40 U/L (Wako Pure Chemical Industries, Ltd, Osaka, Japan). Mouse anti-human-CD3-PerCP, mouse anti-human CD56-APC (Becton Dickinson, USA) and anti-human CD335 (NKp46, Biolegend, USA) were used to detect NK cells by flow cytometry (Becton Dickinson, USA). Data was analyzed using FlowJo7.6 software.

### Statistical Analysis

Data were analyzed by SPSS 19.0 software. All data were presented as mean ± standard deviation (mean ± SD) or median, interquartile interval [median (Q1, Q3)] or percentage. The comparison of data between groups of normal distribution data was completed by analysis of ANNOVA or *t*-test. LSD method was used for multiple comparison if the data between four groups were statistically significant. The comparison of non-normal distribution measurement data was conducted by Kruskal Wallis *H* analysis. Mann Whitney nonparametric *U*-test was used for pairwise comparison if the data between the four groups were statistically significant. Spearman rank correlation test was used to analyze the correlation of variables. *P* < 0.05 was considered statistically significant.

## Results

### Frequency of NK Cells at Baseline

The baseline characteristics of participants are summarized in **Table 1**. At baseline, the frequency of NK cells in pregnant CHB patients were significantly lower than that in normal group [15.30 (12.80, 18.40)], both P < 0.05. Frequency of NK cells in pregnant CHB patients [untreated: 6.90 (4.89, 10.04); treated: 9.42 (6.55, 14.10)] was lower than that in non-pregnant CHB patients (CHB group: 10.60 (6.00, 18.30), *P*=0.001, *P*=0.047, respectively].

**Table 1:** Baseline characteristics of the study population.

Varies	Normal group (n=11)	CHB group (n=26)	Untreated group(n=41)	Treated group (n=46)	*F/t/P* value (Normal vs. CHB)	*F/t/P* value (Normal vs. Untread)	*F/t/P* value (Normal vs. Treated)	*F/t/P* value (CHB vs. Untreated)	*F/t/P* value (CHB vs. Treated)	*F/t/P* value (Untreated vs. Treated)
Age (years)	26.36±1.96	30.42±6.56	29.07±3.46	30.39±4.27	12.775/-2.868/0.013*	5.939/-3.381/0.002*	5.789/-4.660/0.000*	14.096/0.968/0.232	7.678/0.022/0.977	0.938/-1.569/0.174
ALT (U/L)	9.20(7.90,13.10)	25.20 (20.60, 30.90)	15.40 (12.10, 21.15)	19.40 (14.97, 24.02)	3.119/-3.002/0.006*	1.863/-1.389/0.132	4.625/-7.274/0.000*	0.012/1.205/0.233	5.353/2.149/0.042	3.669/0.444/0.650
AST (U/L)	17.20(12.70,18.30)	20.70 (16.40, 24.40)	18.80 (15.50, 22.25)	21.20 (19.30, 24.37)	1.767/-1.284/0.074	1.362/-1.106/0.274	0.230/-3.077/0.003*	0.726/0.828/0.272	6.395/0.842/0.246	4.302/0.057/0.962
HBVDNA (log_10_ IU/ml)	0	8.15±0.37	7.91±0.94	4.31±0.99				0.496/1.118/0.319	18.087/23.029/0.000*	6.751/17.351/0.000*
HBsAg (log_10_ IU/ml)	0	4.77±0.22	4.43±0.59	4.30±0.46				1.034/3.096/0.009*	4.183/5.059/0.000*	1.031/2.147/0.035*
HBeAg(S/CO)	0	1596.64 (1552.68,1637.09)	1640.00 (1523.40,1779.93)	1313.42 (1000.64,1521.29)				5.328/0.198/0.899	17.820/5.224/0.000*	3.375/3.926/0.000*
NK/PBMC(%)	15.30(12.80,18.40)	10.60 (6.00,18.30)	6.90 (4.89,10.04)	9.42 (6.55,14.10)	4.234/0.716/0.436	0.129/5.352/0.000*	0.710/2.624/0.021*	16.475/3.741/0.001*	7.494/1.515/0.047*	3.937/-2.965/0.019*
CD56^dim^(%)	92.52 (90.23, 93.63)	92.81 (87.61, 97.08)	93.03 (87.20, 95.62)	91.33 (87.20, 94.65)	2.945/-0.345/0.749	1.673/0.509/0.535	3.806/0.544/0.774	0.005/1.025/0.199	2.927/1.072/0.398	1.996/-0.508/0.603
CD56^bright^(%)	3.94 (2.50, 7.46)	5.88 (2.60,11.70)	6.97 (4.38,12.80)	8.67 (5.35, 12.80)	4.392/-1.696/0.372	1.565/-1.478/0.054	3.418/-2.803/0.007*	0.431/1.064/0.188	1.177/-1.281/0.394	1.959/0.500/0.581
NKP46+(%)	88.40 (65.50, 94.30)	91.40 (84.05, 94.75)	93.50 (91.75, 95.40)	95.90 (94.40, 97.20)	0.298/-0.702/0.244	30.721/-2.377/0.000*	50.216/-2.913/0.015*	15.943/-2.835/0.002*	23.199/-4.012/0.000*	1.654/-2.395/0.019
NKp46^high^(%)	9.21(7.22,12.60)	7.17 (5.52,13.81)	20.30 (15.85, 29.65)	23.90 (17.00, 33.20)	2.853/-0.508/0.627	8.292/-6.433/0.000*	7.503/-7.641/0.000*	0.535/-3.971/0.000*	0.054/-4.657/0.000*	0.517/-0.394/0.690
NKp46^dim^(%)	76.10 (56.80, 79.00)	80.00 (68.20, 86.29)	79.70 (70.35, 84.15)	76.10 (66.80, 83.00)	0.096/-1.060/0.192	0.958/-1.695/0.101	2.505/-1.616/0.153	2.602/-0.319/0.731	5.186/-0.040/0.964	0.517/0.394/0.728

*P < 0.05 was considered statistically significant.

Compared with the normal group, pregnancy may increase the frequency of NKp46^+^ NK cells in untreated group and treated group (both *P*<0.05). Antiviral therapy during pregnancy could significantly increase the antepartum frequency of NK cells and NKp46^+^ NK cells (both *P*<0.05, untreated vs. treated) (**Table 1**).

### Postpartum ALT, AST and Frequency of NK Cells Before and After Delivery

The levels of ALT and AST increased significantly at 6 and 12 weeks after delivery and decreased at 24 weeks after delivery in HBV infected pregnant women (Intra group comparison, *P*<0.05), but there was no significant difference in the levels of ALT and AST postpartum between the treated and untreated group (*P*>0.05).

In untreated group, the frequencies of NK cells, CD56bright NK cells were significantly increased at 6 and 12 weeks than those before delivery (Intra group comparison, *P*<0.05). In treated group, the frequencies of NK cells, CD56^bright^ NK cells, NKp46^+^ NK cells and NKp46^dim^ NK cells were significantly increased at 6 and 12 weeks than those before delivery (Intra group comparison, *P*<0.05). The frequencies of NK cells and CD56^bright^ NK cells postpartum were increased significantly in treated group than those in untreated group (*P*<0.05). The frequencies of CD56^dim^ NK cells decreased significantly after delivery in treated than those in untreated patients (*P*<0.05). ALT and AST significantly increased after delivery than those before delivery (Intra group comparison, *P*<0.05) (**Table 2**).

**Table 2:** Changes in NK cell frequency and functional molecular expression after delivery in both pregnant groups.

Varies	Untreated group (n = 41)				Treated group (n = 46)			F value/t value/P value	
	0 week	6 week	12 week	24 week	z value/P value	0 week	6 week			12 week	24 week	z value/P value
ALT	15.40 (12.10,21.15)	48.20 (27.10,18.45)	66.20 (34.00,137.70)	31.20 (23.50,50.10)	a1=-4.530/0.000* a2=-3.619/0.000* a3=-3.771/0.000*	19.40 (14.97,24.02)	39.60 (26.62,59.05)	44.60 (31.15,74.35)	28.45 (23.63,36.73)	a1=-5.583/0.000* a2=-5.012/0.000* a3=-3.070/0.002*	b1 = 3.669/0.444/0.650 b2 = 8.795/-1.592/0.120	b3 = 4.720/-1.469/0.153 b4 = 4.505/-1.529/0.139
AST	18.80 (15.50,22.25)	29.80 (21.70,58.60)	34.90 (24.70,80.50)	25.50 (17.00,33.85)	a1=-4.494/0.000* a2=-3.559/0.000* a3=-3.094/0.002*	21.20 (19.30,24.37)	27.30 (22.43,41.37)	33.50 (23.00,45.85)	22.0 (18.57,28.67)	a1=-4.389/0.000* a2=-4.824/0.001* a3=-1.328/0.184	b1 = 4.302/0.057/0.962 b2 = 8.650/-1.562/0.126	b3 = 4.768/-1.309/0.201 b4 = 4.958/-1.428/0.166
NK/PBMC(%)	6.90 (4.89,10.04)	10.60 (7.14,16.20)	10.55 (6.23,16.15)	9.43 (6.62,11.80)	a1=-4.684/0.000* a2=-4.114/0.000* a3=-2.580/0.010*	9.42 (6.55,14.10)	13.35 (10.73,17.95)	13.20 (10.80,20.70)	13.40 (9.82,19.25)	a1=-4.437/0.000* a2=-3.942/0.000* a3=-1.682/0.093	b1 = 3.937/-2.965/0.019* b2 = 1.701/2.177/0.033*	b3 = 0.185/2.118/0.039* b4 = 1.402/2.669/0.013*
CD56^dim^(%)	93.03 (87.20,95.62)	90.82 (85.90,93.30)	90.19 (84.60,94.53)	91.09 (88.75,93.99)	a1=-2.704/0.007* a2 = 4.286/0.000* a3=-3.622/0.000*	91.33 (87.20,94.65)	87.90 (84.38,92.31)	87.50 (80.20,90.50)	87.20 (81.80,90.77)	a1=-3.824/0.000* a2=-4.130/0.000* a3=-1.886/0.059	b1 = 1.996/-0.508/0.603 b2 = 3.100/-1.302/0.197	b3 = 0.144/40.093/0.000* b4 = 1.478/43.034/0.000*
CD56^brigh^t(%)	6.97 (4.38,12.80)	9.18 (6.70,14.10)	9.00 (5.47,15.40)	8.91 (6.01,11.25)	a1=-2.704/0.007* a2=-4.286/0.000* a3=-3.622/0.000*	8.67 (5.35,12.80)	12.60 (9.66,17.15)	12.50 (9.50,19.80)	12.80 (9.23,18.20)	a1=-4.659/0.000* a2=-4.130/0.000* a3=-1.886/0.059	b1 = 1.959/0.500/0.58 b2 = 2.293/2.330/0.023*	b3 = 0.144/-40.093/0.000* b4 = 1.478/-43.034/0.000*
NKP46+(%)	93.50 (91.75,95.40)	95.20 (91.40,97.30)	93.90 (87.08,97.15)	95.90 (93.00,97.55)	a1=-0.617/0.537 a2=-0.171/0.864 a3=-2.296/0.022*	95.90 (94.40,97.20)	97.10 (95.43,97.80)	97.30 (95.60,97.80)	96.95 (94.83,97.58)	a1=-2.109/0.035* a2=-2.584/0.010* a3=-1.735/0.083	b1 = 1.654/-2.395/0.019* b2 = 5.015/1.805/0.081	b3 = 27.361/3.224/0.003* b4 = 1.642/0.922/0.365
NKp46^high^(%)	20.30 (15.85,29.65)	18.20 (12.60,24.70)	19.15 (14.63,25.73)	17.40 (15.35,21.50)	a1=-1.921/0.055 a2=-3.043/0.002* a3=-2.533/0.011*	23.90 (17.00,33.20)	18.40 (12.05,22.63)	16.70 (11.40,21.50)	14.10 (11.43,17.93)	a1=-4.095/0.000* a2=-3.740/0.000* a3=-2.701/0.007*	b1 = 0.517/-0.394/0.690 b2 = 2.281/-1.313/0.194	b3 = 0.461/-1.271/0.210 b4 = 0.489/-1.720/0.098
NKp46^dim^(%)	79.70 (70.35,84.15)	81.80 (75.30,87.40)	80.85 (74.28,85.38)	82.60 (78.50,84.65)	a1=-1.921/0.055 a2=-3.043/0.002* a3=-2.533/0.011*	76.10 (66.80,83.00)	81.60 (77.38,87.95)	83.30 (78.50,88.60)	85.90 (82.08,88.58)	a1=-4.095/0.005* a2=-3.740/0.000* a3=-2.701/0.007*	b1 = 0.517/0.394/0.728 b2 = 2.281/1.313/0.194	b3 = 0.461/1.271/0.210 b4 = 0.489/1.720/0.098

1. 0 week=before delivery 6 week=6 week after delivery 12 week=12 week after delivery 24 week=24 week after delivery.

2. Comparsion within groups: a1: 0 week vs 6 week a2: 0 week vs 12 week a3: 0 week vs 24 week.

3. Comparsion among groups: b1: 0 week vs 0 week b2: 6 week vs 6 week b3: 12 week vs 12 week b4: 24 week vs 24 week.

4. *P < 0.05 was considered statistically significant.

### Correlation Between Postpartum ALT Level and NK Frequency in Pregnant CHB Women

**Figure 1:**
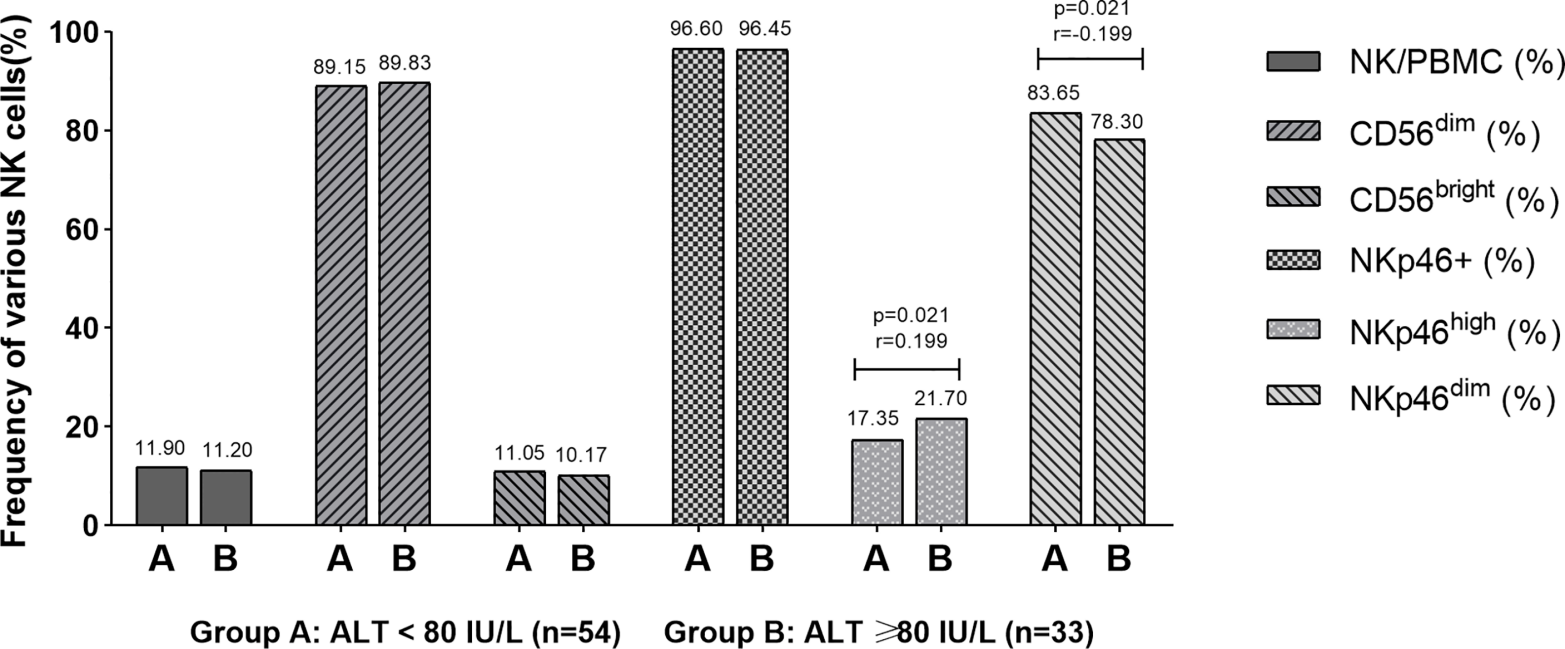
Correlation between postpartum ALT level and NK frequency. The results showed that the postpartum ALT level was weak positive correlated with NKp46^high^ frequency (*r* = 0.199) and weak negative correlated with NKp46^dim^ frequency (*r* = -0.199).

In order to explore the relationship between postpartum hepatitis and NK cells in pregnant women, 87 pregnant women were further divided according to the level of ALT (54 cases in group A with ALT < 80 U/L and 33 cases in group B with ALT 80≥U/L). The correlation between ALT level and frequency of NK cells was analyzed with Spearman rank correlation test. The NKp46^high^ frequency in patients with postpartum ALT ≥ 80 U/L was higher than that in patients with ALT<80 U/L (*P* = 0.021), and the NKp46^dim^ frequency in patients with postpartum ALT ≥ 80 U/L was lower than that in patients with ALT<80 U/L (*P* = 0.021). The results showed that the postpartum ALT level was weak positive correlated with NKp46^high^ frequency (*r*=0.199) and was weak negative correlated with NKp46^dim^ frequency (*r*= -0.199) (**Figure 1**).

## Discussion

NK cells, one of the important effector cells in the body’s antiviral immunity, account for 5%-10% of the total number of lymphocytes in human peripheral blood. Chronic HBV infection leads to decrease in the number of NK cells and function (5). NK cells can be divided into two categories according to the number of CD56 molecules expressed on the cell surface. More than 90% are CD56^dim^ NK cells, which mainly mediates the cytotoxicity and killing effect of NK cells, while less than 10% are CD56^bright^ NK cells, which secretes some important immunomodulatory cytokines and has weak cytotoxic activity (6).

Our results suggested that the frequency of NK cells was reduced under the condition of pregnancy. Studies have reported that NK cells play a major role in the occurrence of CHB (7–10). HBV virus can damage and inhibit the function of NK cells (11). The frequency of CD56^bright^ NK cells in peripheral blood of patients with CHB treated with nucleoside increased after reaching HBV-DNA inhibition and low serum HBsAg level (12). Stelma et al. (13) found that polyethylene glycol α interferon combined with adefovir dipivoxil antiviral treatment could increase the frequency and absolute number of CD56^bright^ NK cells in peripheral blood, and decrease the frequency and absolute number of CD56^dim^ NK cells, which is consistent with our results.

NKp46 is a specific cytolytic receptor expressed in mature NK cells, which is divided into NKp46^high^ and NKp46^dim^ subtypes according to the expression of NKp46 receptor. NKp46 can activate NK cells and participate in the recognition and cytotoxicity of target cells without antigen pre-stimulation (14–18). NKp46 is the first way to resist virus infection in the early stage line of defense. In this study, the frequency of NKp46^+^ NK cells was significantly increased in pregnant women, indicating that pregnant may stimulate the expression of NKp46 molecules. We further found that antiviral therapy in the third trimester of pregnancy can significantly increase the expression of NKp46 molecules.

We further investigated the changes of NK cells in pregnant women with CHB and observed that the frequency of NK cells increased significantly at 6 weeks postpartum and maintained a steady state during 6-24 weeks postpartum. Our previous retrospective study (3) showed an increase of ALT level in normal pregnant women soon after delivery, which is consistent with the increase of NK cell frequency. It speculates that the recovery of postpartum immune function will produce a certain immune response to liver cells and lead to the emergence of liver inflammation, which may be the reason for the increase of ALT level in normal women after delivery. However, for HBV infected pregnant women, the recovered immune function further produces immune response to HBV virus, resulting in the re increase of ALT level in 6-12 weeks postpartum, which may be the reason for the bimodal change of ALT level in HBV infected pregnant women in our retrospective study (3). This study also showed that antiviral treatment could significantly improve the frequency of NK cells in postpartum women. The frequency of NK cells in the treated group was higher than that in the women without antiviral treatment. It also reminds us that the decrease of HBV DNA level and HBeAg level may reduce the effect of HBV virus and HBeAg on NK cells.

In conclusion, the results of this study indicate that HBV infection and pregnancy may reduce the frequency of NK cells, while antiviral treatment during pregnancy could significantly increase the frequency of NK cells. Postpartum hepatitis may be related to the immune injury caused by change of NK cell frequency and HBV infection. However, due to the small number of cases in this study, the conclusions need be further verified.

## Data Availability Statement

The raw data supporting the conclusions of this article will be made available by the authors, without undue reservation.

## Ethics Statement

The studies involving human participants were reviewed and approved by the ethics committee of Beijing Ditan Hospital, Capital Medical University (JDL-2017-004-01). The patients/participants provided their written informed consent to participate in this study.

## Author Contributions

WY, ML, and YX contributed to the study design. CR, SX, and HH contributed to the data analysis. CR, SX, HH, WY, ML, and YX contributed to the recruitment, enrolment, and assessment of participants, as well as data collection. TJ, WD, FS, and XB contributed to following up with the patients. FW, CR, YL, LY, and ZZ managed all aspects of laboratory support. FW wrote the first draft of the manuscript. WY, ML, and SX revised the manuscript and is the guarantor of the article. All authors approved the final version of the manuscript.

## Funding

This project was supported by the Beijing Hospitals Authority Clinical medicine Development of Special Funding Support (No. XMLX 201706 and XMLX 202127), National Science and Technology Major Project of China (No. 2017ZX10201201-001-006 and 2017ZX10201201-002-006, and 2018ZX10715-005-003-005), Special Public Health Project for Health Development in Capital (2021-1G-4061 and 2022-1-2172), the Digestive Medical Coordinated Development Center of Beijing Hospitals Authority (No. XXZ0302 and XXT28), Beijing Science and Technology Commission (No. D161100002716002), and Beijing Municipal Science & Technology Commission (No. Z151100004015122).

## Conflict of Interest

The authors declare that the research was conducted in the absence of any commercial or financial relationships that could be construed as a potential conflict of interest.

## Publisher’s Note

All claims expressed in this article are solely those of the authors and do not necessarily represent those of their affiliated organizations, or those of the publisher, the editors and the reviewers. Any product that may be evaluated in this article, or claim that may be made by its manufacturer, is not guaranteed or endorsed by the publisher.

## References

[1] Huang Y Li L Sun X Lu M Liu H Tang G. Screening of Pregnant Women for Hepatitis Bvirus Surface Antigen (HBsAg) and Subsequent Management, Qiandongnan Prefecture, Guizhou, China, 2010. Vaccine (2013) 31:J62–5. doi: 10.1016/j.vaccine.2013.05.103 24331022

[2] Yao H Hui Y. Prevention Strategies of Mother-to-Child Transmission of Hepatitis B Virus (HBV) Infection. Pediatr Investig (2020) 4(2):133–7. doi: 10.1002/ped4.12205 PMC7331440 32851357

[3] Yi W Pan CQ Li MH Wan G Lv YW Liu M. The Characteristics and Predictors of Postpartum Hepatitis Flares in Women With Chronic Hepatitis B. Am J Gastroenterol (2018) 113:686–93. doi: 10.1038/s41395-018-0010-2 29487412

[4] European Association For The Study Of The Liver. EASL Clinical Practice Guidelines: Management of Chronic Hepatitis B Virus Infection. J Hepatol (2012) 57:167–85. doi: 10.1016/j.jhep.2012.02.010 22436845

[5] Wu J Han M Li J Yang X Yang D. Immunopathogenesis of HBV Infection. Adv Exp Med Biol (2020) 1179:71–107. doi: 10.1007/978-981-13-9151-4_4 31741334

[6] X B Huang H Gao S Xu X. Echinococcus Multilocularis Induces Surface High Expression of Inhibitory Killer Immunoglobulin-Like Receptor on Natural Killer Cells. Allergol Immunopathol (2021) 49:78–86. doi: 10.15586/aei.v49i5.465 34476926

[7] Wijaya RS Read SA Schibeci S Han S Azardaryany MK van der Poorten D. Expansion of Dysfunctional CD56-CD16+ NK Cells in Chronic Hepatitis B Patients. Liver Int (2021) 41:969–81. doi: 10.1111/liv.14784 33411395

[8] Bonorino P Ramzan M Camous X Dufeu-Duchesne T Thélu M-A Sturm N. Fine Characterization of Intrahepatic NK Cells Expressing Natural Killer Receptors in Chronic Hepatitis B and C. J Hepatol (2009) 51:458–67. doi: 10.1016/j.jhep.2009.05.030 19596474

[9] Du Y Anastasiou OE Strunz B Scheuten J Bremer B Kraft A. The Impact of Hepatitis B Surface Antigen on Natural Killer Cells in Patients With Chronic Hepatitis B Virus Infection. Liver Int (2021) 41:2046–58. doi: 10.1111/liv.14885 33794040

[10] Zhao J Li Y Jin L Zhang S Fan R Sun Y. Natural Killer Cells Are Characterized by the Concomitantly Increased Interferon-γ and Cytotoxicity in Acute Resolved Hepatitis B Patients. PloS One (2012) 7:e49135. doi: 10.1371/journal.pone.0049135 23133672 PMC3486810

[11] Chen Y Wei H Sun R Tian Z. Impaired Function of Hepatic Natural Killer Cells From Murine Chronic HBsAg Carriers. Int Immunopharmacol (2005) 5:1839–52. doi: 10.1016/j.intimp.2005.06.004 16275620

[12] Liu N Liu B Zhang L Li H Chen Z Luo A. Recovery of Circulating CD56(dim) NK Cells and the Balance of Th17/Treg After Nucleoside Analog Therapy in Patients With Chronic Hepatitis B and Low Levels of HBsAg. Int Immunopharmacol (2018) 62:59–66. doi: 10.1016/j.intimp.2018.06.043 29990695

[13] Stelma F de Niet A Tempelmans Plat-Sinnige MJ Jansen L Takkenberg RB Reesink HW. Natural Killer Cell Characteristics in Patients With Chronic Hepatitis B Virus (HBV) Infection Are Associated With HBV Surface Antigen Clearance After Combination Treatment With Pegylated Interferon Alfa-2a and Adefovir. J Infect Dis (2015) 212:1042–51. doi: 10.1093/infdis/jiv180 25791117

[14] Li W Jiang Y Wang X Jin J Qi Y Chi X. Natural Killer P46 Controls Hepatitis B Virus Replication and Modulates Liver Inflammation. PloS One (2015) 10:e0135874. doi: 10.1371/journal.pone.0135874 26291078 PMC4546267

[15] Mai C Fukui A Takeyama R Yamamoto M Saeki S Yamaya A. NK Cells That Differ in Expression of NKp46 Might Play Different Roles in Endometrium. J Reprod Immunol (2021) 147:103367. doi: 10.1016/j.jri.2021.103367 34464905

[16] Gur C Porgador A Elboim M Gazit R Mizrahi S Stern-Ginossar N. The Activating Receptor NKp46 Is Essential for the Development of Type 1 Diabetes. Nat Immunol (2010) 11:121–8. doi: 10.1038/ni.1834 20023661

[17] Sivori S Vitale M Morelli L Sanseverino L Augugliaro R Bottino C. P46, a Novel Natural Killer Cell-Specific Surface Molecule That Mediates Cell Activation. J Exp Med (1997) 186:1129–36. doi: 10.1084/jem.186.7.1129 PMC2211712 9314561

[18] Moretta L Pietra G Montaldo E Vacca P Pende D Falco M. Human NK Cells: From Surface Receptors to the Therapy of Leukemias and Solid Tumors. Front Immunol (2014) 5:87. doi: 10.3389/fimmu.2014.00087 24639677 PMC3945935

